# Reconstruction of Chronic Injured Distal Tibiofibular Syndesmosis with Autogenous Tendon Graft: A Systematic Review

**DOI:** 10.1155/2021/3182745

**Published:** 2021-02-01

**Authors:** Han-Lin Xu, Yu-Jie Song, Ying-Hui Hua

**Affiliations:** Department of Sports Medicine, Huashan Hospital, Fudan University, No. 12 Urumqi Middle Rd., Shanghai 200040, China

## Abstract

**Background:**

Chronic injuries of the distal tibiofibular syndesmosis are common in patients who fail to receive adequate diagnosis and timely treatment. Reconstruction of the distal tibiofibular syndesmosis with an autogenous tendon graft in these patients is effective, although relatively rarely reported.

**Purpose:**

To investigate clinical outcomes of syndesmosis reconstruction with an autogenous tendon graft for chronic injuries of the distal tibiofibular syndesmosis by reviewing the current literature.

**Methods:**

An English literature search was conducted in the MEDLINE, CENTRAL, and Cochrane databases to identify published studies up to October 2017. Preset inclusion and exclusion criteria were applied to identify all eligible articles.

**Results:**

Five studies (all with level IV evidence) that included a total of 51 patients who underwent reconstruction with an autogenous tendon graft were identified. It was reported that the symptoms were relieved postoperatively, including obviously improved functional outcomes and restoration of motions and exercise capacity. The mean American Orthopedic Foot and Ankle Society scale score of 16 patients was 53 preoperatively and 89 postoperatively. The visual analogue scale score of 14 patients decreased from 82.4 preoperatively to 12.6 postoperatively. A total of 5 (9.8%) complication cases were reported.

**Conclusion:**

Reconstruction of the distal tibiofibular syndesmosis with an autogenous tendon for chronic syndesmosis injury showed a good therapeutic effect in terms of both subjective symptoms and objective evaluation scores. The interosseous ligament could be an appropriate reconstruction target in the treatment of chronic syndesmosis injury.

## 1. Introduction

Distal tibiofibular syndesmosis injuries are frequently misdiagnosed or not treated timely; however, the prevalence has been underestimated [1]. Failure to recognize an acute syndesmosis injury might aggravate the condition and cause its progression to the final chronic stage, which is defined as an injury course of >6 months [2–5]. Herman et al. reported that the prevalence of distal tibiofibular injuries was 1–11% among patients with ankle sprains and that 40% of these patients with syndesmosis injuries still have complaints of ankle instability at 6 months after the occurrence of ankle sprain [6].

Surgical treatment methods for chronic injury of the distal tibiofibular syndesmosis include debridement, arthrodesis, screw fixation, suture-button repair, and graft reconstruction [7, 8]. Although there are various surgical methods of surgical reconstruction for chronic injury of the distal tibiofibular syndesmosis, no consensus has been reached [2, 7, 9]. Studies of comparison between screw fixation and suture-button have been performed, and dynamic repair is considered to be critical to reduce failure rates and for optimal outcomes. However, few researches have been done on autogenous tendon graft reconstruction of distal tibiofibular syndesmosis as a common treatment for chronic ligament injuries [10].

The relationship between articular micromovement and stabilization was mostly balanced by ligamentous tissues [8]. Graft reconstruction has unique advantages in treating chronic joint injuries and restoring range of motion, especially when patients lack sufficient ligamentous tissue or have abnormal motions of the syndesmosis. Here, we performed this review to summarize the outcomes of syndesmosis reconstruction with an autogenous tendon graft for chronic syndesmosis injuries by reviewing the current literature. We hypothesized that this treatment could achieve satisfactory results and even show better outcomes than other surgical methods in the long-term follow-up.

## 2. Materials and Methods

The present study adhered to the PRISMA (Preferred Reporting Items for Systematic Reviews and Meta-analyses) criteria [10, 11].

### 2.1. Search Strategy

The research was based on English articles obtained using the MEDLINE, CENTRAL, and Cochrane databases. The databases were searched using the following query terms: “((((syndesmosis OR syndesmotic) AND (tibiofibular OR distal)) AND chronic) AND (reconstruction OR ligamentoplasty) AND ((injury OR disruption) OR instability)” in all fields (through October 2017). An additional search of references of the selected studies was performed manually, and another 5 relevant articles were found. The search yielded 140 reports in total, and 133 abstracts were left for review after the removal of duplicates.

### 2.2. Inclusion and Exclusion Criteria

Inclusion criteria for the studies were as follows: (i) retrospective design investigating the clinical results of syndesmosis reconstruction with a tendon graft for chronic tibiofibular syndesmosis injury, (ii) reporting functional evaluation results (including AOFAS, VAS, Karlsson score SF-36, Maryland, and Weber score) before and after surgery, and (iii) mean follow-up duration of >12 months. Exclusion criteria were as follows: (i) not published in English, (ii) investigating syndesmosis reconstruction without a tendon graft, (iii) cadaveric or biomechanical studies based on computer models and other laboratory reports, and (iv) editorials, expert opinions, case reports, and other descriptive studies.

### 2.3. Study Selection

**Figure 1:**
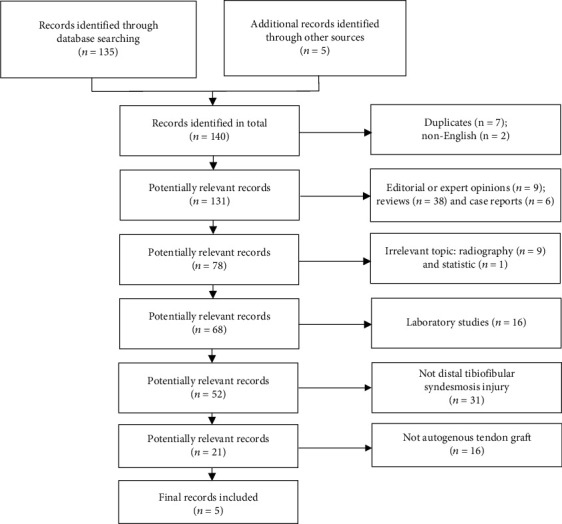
Flow diagram of study selection.

A total of 133 potentially eligible reports were reviewed by scanning the titles and abstracts. Only studies published in English were considered. Then, 81 reports with irrelevant topics or that met the exclusion criteria and 31 reports focusing on some other joint injuries or dysfunction were dropped. The full text of an article was checked when the title or abstract mentioned chronic injuries of the distal tibiofibular syndesmosis. Another 16 studies were eliminated because of the application of screw fixation, suture-button repair, or joint fusion instead of an autograft tendon. Finally, 5 studies with a total of 51 enrolled patients were included in this systematic review (Figure 1).

### 2.4. Data Extraction and Statistical Analysis

Descriptive data such as number of patients, mean age of patients, mean follow-up duration, mean interval between sprains and surgeries, postoperative functional evaluation, autogenous graft used, and time from surgery to initial weight bearing were collected. All statistical analyses were performed by using SPSS 13.0.

Clinical symptoms, injury classification, symptom or radiographic improvements, function evaluation outcomes, and surgical complications were also summarized. Mean values of all available quantitative data were calculated and weighted using the number of enrolled patients in each study.

### 2.5. Quality Assessment

The Coleman methodology score was used to assess the risks of bias and quality of research [12] (Table 1).

## 3. Results

A total of 5 studies met the inclusion and exclusion criteria. The descriptive data of these studies are shown in Table 2. The descriptive conclusions of the 5 articles are summarized in Table 3.

**Figure 2:**
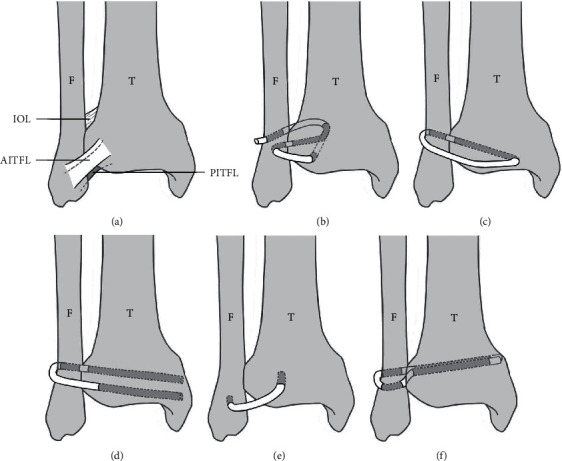
Comparisons of normal anatomy of syndesmosis and graft reconstruction methods on the right ankle joint: (a) Illustrated the normal anatomical position of AITFL, IOL, and PITFL; (b–f) illustrated surgical methods (the direction and location of the tunnels and grafts) in 5 studies, respectively. T: tibia; F: fibula; AITFL: anterior inferior tibiofibular ligaments; IOL: interosseous ligaments; PITFL: posterior inferior tibiofibular ligaments; grey lines: grafts.

In a retrospective cohort study, Grass et al. introduced a 3-point fixation method with a split peroneus longus tendon graft in 16 patients with chronic syndesmosis injuries [13]. Reconstruction was performed with a split peroneus longus tendon graft that was inserted into 3 canals, mimicking the location and direction of the normal anterior inferior tibiofibular ligament (AITFL), posterior inferior tibiofibular ligament, and interosseous ligament (IOL) (Figure 2(b)). The graft was fixed with a 3.5 mm cancellous screw. The Karlsson scoring system and coronal computed tomography were used to evaluate the surgical outcomes. Fifteen patients achieved pain relief, and all patients regained ankle stability after an average of 16.4 months follow-up. The mean postoperative Karlsson score reached 88 points (range, 70–100). The postoperative total clear space and tibiofibular distance were markedly reduced by an average of 45.7% and 45.2%, respectively, on coronal computed tomography, whereas the mean total fibular overlapping increased by 80.6%.

Zamzami and Zamzam conducted a retrospective study reviewing 11 patients with isolated chronic syndesmosis injury [14]. All recruited patients only had ligament injuries without bony fractures and failed conservative treatments such as anti-inflammatory medication or physiotherapy. Open reconstructive surgeries were performed in all patients by drilling 1 tunnel from the posterolateral fibula to the anterolateral tibia 1 cm above the ankle joint. A 10 cm semitendinosus tendon was harvested. It was threaded through the tunnel, sutured circularly, and fixed with a 3.5 mm screw 1 cm above the tunnel to assist syndesmosis reduction (Figure 2(c)). The postoperative mean West Point Ankle Score System score was 95.4. Ten patients achieved complete resolution, whereas 1 patient achieved only pain relief.

Morris et al. [15] investigated another method for reconstructing the anterior tibiofibular ligament. There were 8 patients with chronic syndesmosis instability enrolled in the present case series. A semitendinosus autograft was used to reconstruct the AITFL and IOL. This reconstruction was finished with 2 tunnels drilled 2 cm above the tibial plafond, in which one passed from the posterolateral fibula to the anterior tibia and the other was drilled below the first one, through the anterior tibia in the same direction. Then, the tendon was threaded through 2 tunnels from the medial to lateral aspects and fixed with a 15 mm interference metal screw (Figure 2(d)). The latest follow-up suggested conspicuous elimination of ankle pain and instability. The postoperative mean American Orthopedic Foot and Ankle Society (AOFAS) scale score was 85.4. The visual analogue scale (VAS) score decreased from 73 preoperatively to 19 postoperatively. No serious complications were reported in the article.

Yasui et al. introduced a single-tunnel AITFL reconstruction procedure and collected and compared the preoperative and postoperative data of 6 patients with tibiofibular syndesmosis disruption [16]. In this specific surgical method, 2 bone tunnels (diameter, 5.5 mm; depth, 25 mm) were established on the anterolateral tibia and anterior fibula, which are the origin and the termination of the AITFL, respectively. An interference screw was applied to secure the autogenous gracilis tendon in 2 canals (Figure 2(e)). At the latest follow-up, the significantly higher AOFAS score and lower VAS score of patients than the preoperative values represented an optimistic outcome.

Colcuc et al. compared AITFL suture, ligament repair with periosteal flaps, and ligamentous reconstruction with an autogenous plantaris tendon graft [7]. They divided 32 patients into 3 groups according to different grades of fibular dislocation. For the purpose of this review, only the last method was analyzed. Ten patients with a >2.5 mm fibular dislocation underwent AITFL and IOL reconstruction. Two fibula tunnels and 1 tibia tunnel were drilled at the level of the AITFL and IOL. A Bio-Tenodesis screw (Arthrex Inc., Naples, FL) was inserted into the tibial tunnel to fix the free end of the graft (Figure 2(f)). The changes in the AOFAS score, from a preoperative score of 53 ± 13 points (mean ± standard deviation) to a postoperative score of 86 ± 5 points, and in the Weber score, from a preoperative score of 12 ± 3 points to a postoperative score of 2 points, also revealed a significant clinical improvement.

The pooled results of the 5 studies showed satisfactory outcomes of ligament reconstruction. Although Yasui et al. [16] and Colcuc et al. [7] did not evaluate clinical symptoms, there were 34 cases of postoperative pain relief among 35 patients from the other 3 studies [13–15]. Zamzami and Zamzam [14] and Morris et al. [15] reported 18 (94.7%) cases of functional recovery in a total of 19 patients. The quantitative follow-up, which spanned 27.8 months on average, showed optimistic outcomes. In 2 studies [7, 16] with 16 patients, the average AOFAS score changed from 53 preoperatively to 89 postoperatively. The VAS scores of 14 patients in 2 studies [15, 16] decreased from 82.4 preoperatively to 12.6 postoperatively at the last follow-up. Moreover, the reconstruction effectively helped 7 of 8 (87.5%) patients in regaining their confidence to return to work and exercise. From this perspective, reconstruction was especially suitable for chronic injuries [13]. Yasui et al. reported that pronation external rotation stage IV injury with residual remarkable opening of the anterior part could be successfully treated using anatomical reconstruction of the AITFL with an autogenous gracilis tendon [16].

## 4. Discussion

The foremost findings of this systematic review are the potential advantages of ligament reconstruction for chronic syndesmosis injuries and the underestimated value of IOL reconstruction in the rehabilitation of patients with chronic syndesmosis injuries.

The distal tibiofibular syndesmosis consists of the AITFL, posterior inferior tibiofibular ligament, inferior transverse ligament, and IOL [2, 17, 18]. These ligaments are considered responsible for 9–35% of ankle joint stability, which has a strong link to long-term articular function [14, 19–21]. These ligaments function by limiting the relative position between the fibula and tibia and preventing the lateral shift of the talus and fibula when the malleolus is dorsiflexed [22]. Pronation and external rotation injuries are considered the major injury mechanism in patients with syndesmosis injuries, usually causing lateral shift of the talus and fibula, which finally cause ankle instability [2, 8, 23, 24]. Ramsey and Hamilton proved that the tibiotalar contact area was decreased by 42%, and the force exerted on the remaining surface increased by 30% if the talus shifted laterally for even only the initial 1 mm [25]. The gold standard method of evaluating syndesmosis injuries is inserting an arthroscopic probe between the tibia and fibula, and a gap wider than 2 mm was defined as a convincing indication for surgery [7, 24, 26, 27]. [7, 23, 25, 26] As a result, the probability of secondary arthritis significantly increased, as reflected by symptoms including sustained diffuse pain around the lateral ankle, lateral ankle swelling, and sense of giving way [13].

Various approaches have been used for acute syndesmosis injury, including debridement, arthrodesis, screw fixation, suture-button repair, and graft reconstruction. However, not all these strategies can be applied to chronic syndesmosis instability, and the treatment of chronic injuries is more challenging than that of acute injuries [8, 21]. Screw fixation is a widely used surgical treatment for acute syndesmosis injuries; however, long-term follow-up revealed some complications, such as screw loosening, breakage, and even reoperation [28, 29]. Recently, a series of dynamic reconstruction devices were applied more frequently to meet the patients' desire to return to work and sports, including suture-button wire cerclages or elastic hook plates characterized by allowing of physiological movement of joint [30]. As a result of comparison, the conduction of anatomical and flexible surgical methods has become an ideal option for more surgeons.

The distal tibiofibular syndesmosis has been considered a physiological micromotion joint [8, 31, 32]. Anatomical ligamentous reconstruction for chronic syndesmosis injuries is the key to maintaining articulation stability and avoiding alterations of physiologic biomechanics [24, 31, 33, 34]. Nevertheless, previous studies noted that this characteristic could not be reconstructed using simple screw fixation or arthrodesis [21, 26, 35]. Johnson et al. reported that screw fixation does not allow normal motion of the syndesmosis during healing because the screw might break or become loosened [26]. Previous other studies reported that simple screw fixation is likely to cause joint stiffness [10, 36]. Lui believed that arthrodesis is an unfavorable method for patients with advanced ankle degeneration that would deteriorate the syndesmosis motion in the long term [37]. Olson et al. used arthrodesis in patients with syndesmosis injury, which yielded satisfactory symptomatic outcomes; however, no improvement was seen in the AOFAS motion subscale score postoperatively [38]. However, this review revealed that a flexible graft could restore this characteristic and could decrease the possibility of postoperative ankle stiffness, as the reconstruction effectively helped 7 of 8 patients to return to work and exercise [15].

In terms of postoperative complications, graft reconstruction showed consistent outcomes in the long term [13, 14]. No graft rejections or ruptures were observed in all 51 patients in a mean follow-up of 24.7 months, and only 6 of 51 (11.8%) patients who underwent graft reconstruction developed superficial wound infection with none of them needing further surgeries [7, 13–16]. Postoperative adverse events were more frequently reported with other surgical methods [21, 39]. Zhang et al. reported implant failure after screw fixation in 48 (30.9%) patients and malreduction after surgery in 12 (12.6%) patients [10]. Neary et al. reported that the screw removal or failed syndesmotic screw rates ranged from 5% to 52% in 6 studies [40]. With respect to suture-button repair, rates of failed implantation requiring removal ranged from 0% to 10% in 4 studies [40]. Xie et al. reported that the rewidening of the distal syndesmosis space after screw removal and the formation of scar tissue might affect the complication rates in patients undergoing screw fixation [21]. It was reasonable to speculate, based on the available studies, that ligament reconstruction leads to fewer complications.

The position and direction of ligament reconstruction are also intensely debated. As the syndesmosis is described as a tight connection of the tibia and fibula held together by a strong membrane or ligament composed of connective tissue, the term “syndesmosis injury” refers to injury of the syndesmosis ligaments [6, 8]. Thus, treatments of syndesmosis injuries are aimed at reconstructing the ligamentous anatomical conditions and biomechanical characteristics. The location and direction of tunnels chosen by the included articles were similar to those of the AITFL and IOL (Figure 2). Of the 5 methods, IOL reconstruction was performed in 4 methods, and AITFL reconstruction was performed in 4 methods.

In a previous study, the surgeons considered AITFL injury as the primary crucial mechanism of syndesmosis injuries [41]. Ogilvie-Harris et al. conducted a cadaveric dynamical study and found that the rate of relative importance of the individual syndesmosis ligaments to syndesmotic stability was 22% for IOL and 35% for AITFL [42]. However, the percentage was concluded using divergent cutting sequences of syndesmosis ligaments in a small sample size of 8 ankles. Therefore, the proportions of significance of the ligaments were not convincing. Hoefnagels et al. performed another study comparing the biomechanical importance of the IOL and AITFL [43]. The results showed that the strength of the IOL in terms of load-carrying capacity (822 ± 298 N) was significantly greater than that of the AITFL (625 ± 255 N). The IOL was also reported to act as a buffer for the transfer of axial tibial load from the tibia to the fibula [44]. This prompted us to verify whether the studies had underestimated the importance of the IOL in limiting the pronation of the fibula and in guaranteeing the congruency of the articulation.

Another clinical basis for this argument is that isolated IOL disruption without AITFL injuries could also lead to diastasis between the fibula and tibia [6, 27]. The ATIFL is also reported to be the weakest of the 4 syndesmosis ligaments, and the ligament that can most easily yield external rotation forces [6]. Hermans et al. considered the IOL as a ligament that maintains the micromotion characteristics of the ankle, as a buffer that neutralizes forces in walking and in stabilizing the ankle joint during loading [6, 45, 46]. As the actual importance of the IOL in maintaining fibula stability is still controversial, more evidence should be collected in the future to confirm the necessity of IOL reconstruction [2, 43].

The present study has some limitations. (i) All included studies lacked randomization and a control group for comparison, which degraded the level of evidence. More level I studies are needed for further analyses. (ii) The diversity of operation methods and postoperative regimen in different studies made it difficult to pool the outcomes and draw a convincing conclusion. A well-recognized integrated postoperative evaluation list can be created for future use. (iii) The number of studies and patients was insufficient, and the postoperative evaluation periods were short. The small number of studies and patients added a random error, and the insufficient follow-up period resulted in the lack of long-term prognostic evaluation. Future studies including a larger study cohort in both the experimental and control groups as well as with a postoperative observation of at least 3 years are needed.

## 5. Conclusion

Reconstruction of the distal tibiofibular syndesmosis with an autogenous tendon for chronic syndesmosis injury showed a good therapeutic effect in terms of both subjective symptoms and objective evaluation scores. The IOL could be an appropriate reconstruction target in the treatment of chronic syndesmosis injury.

## Figures and Tables

**Table 1:** Coleman methodology scores for selected studies.

Methodology criterion (min–max)	Grass et al. [13]	Zamzami and Zamzam [14]	Morris et al. [15]	Yasui et al. [16]	Colcuc et al. [7]
Part A					
(1) Study size (0-10)	0	0	0	0	0
(2) Mean follow-up (0-5)	2	5	5	5	2
(3) Number of procedures (0-10)	10	10	10	10	10
(4) Type of study (0-15)	0	0	0	0	0
(5) Diagnostic certainty (0-5)	5	5	5	5	5
(6) Description of surgical technique (0-5)	5	5	5	5	5
(7) Rehabilitation and compliance (0-10)	10	10	10	10	10
Part B					
(1) Outcome criteria (0-10)	10	10	7	8	10
(2) Outcome assessment (0-15)	3	6	6	6	11
(3) Selection process (0-15)	5	5	5	5	5
Total Coleman methodology score (0-100)	50	56	53	54	58

**Table 2:** Descriptive data of the included studies.

Study (years)	Number of patients	Mean age (years)	Follow-up (months)	Functional evaluation	Mean intervals (months)	Autogenous graft usage	Initial postoperative weight bearing (weeks)
Grass et al. [13] (2003)	2 men, 14 women	40	16.4 (range from 13 to 29)	Karlsson score	14 (besides two chronic patients)	Peroneus longus tendon	1
Zamzami and Zamzam [14] (2009)	11 men	31.5 (range from 19 to 44)	37 (range from 24 to 60)	West Point Ankle Score System	56 (range from 24 to 120)	Semitendinosus tendon	9-11
Morris et al. [15] (2009)	8 patients	33.9 (range from 17 to 46)	39 (range from 9 to 86)	AOFAS, VAS, SF-36, Maryland	13 (range from 3 to 28)	Semitendinosus tendon	2
Yasui et al. [16] (2011)	5 men, 1 woman	35 (range from 19 to 56)	44 (range from 31 to 50)	AOFAS, VAS	12 (range from 10 to 27)	Gracilis tendon	4
Colcuc et al. [7] (2016)	10 patients	41 (range from 18 to 71)	17	AOFAS, Weber score	11 (range from 7 to 15)	Plantaris longus tendon	8

**Table 3:** Diagnosis and treatment information of the included studies.

Study (years)	Clinical symptoms	Injury classification	Symptoms/radiograph improvements	Function evaluation outcome	Surgical complications
Grass et al. [13] (2003)	Diffuse pain, persistent swelling of the ankle region	14 patients had a PER stage III and IV injury, and 2 patients had a pronation abduction stage II injury	The mean TCS decreased from 7.0 (range, 6–9) mm to 4.8 (range, 2–5) mm. The mean TFO increased from 3.6 (range, 2–5) mm to 6.5 (range, 5–8) mm. The tibiofibular distance (CT-TFD) decreased from 6.2 (range, 4.1–8.1) mm to 3.0 (range, 1.8–4.5) mm postoperatively	The mean Karlsson score at the time of follow-up was 88 (range from 77 to 100)	1 breakage of the screw and the clinical but radiological result was unaffected
Zamzami and Zamzam [14] (2009)	Chronic unilateral ankle pain, sense of ankle instability in 9, ankle stiffness in 2, and occasional attacks of ankle locking in 3	7 patients had twisting trauma (5 were external rotation injuries); 1 patient got direct trauma; 3 patients could not remember the mechanism of injury	10 patients had full recovery with complete resolution of their previous symptoms. Only 1 patient had persistent limitation of ankle dorsiflexion with pain free. Average amount of radiographic diastasis between tibia and fibula in millimeter decreased from 4.7 to 1.3	West Point Ankle Score System, the average score for the patients was 95.4 points (range, 87–100)	2 superficial stitch abscesses
Morris et al. [15] (2009)	Anterolateral pain, recurrent ankle swelling, and feeling of instability	NR	Clinically, the patients all reported improved symptoms, and no further symptoms of instability. 7 of 8 returned to sports and to their previous jobs	73 pre-19 post (VAS)	1 superficial infection
Yasui et al. [16] (2011)	Unsolved persistent pain after initial treatment	PER stage IV injury with residual remarkable opening of the anterior part	NR	53 pre-95 post (AOFAS), 95 pre-4 post (VAS)	No complications
Colcuc et al. [7] (2016)	NR	NR	NR	53 pre-86 post (AOFAS)	1 superficial infection; 2 suture granulomas

TCS: total clear space; TFP: total fibular overlapping: TFD: tibiofibular distance: NR: not reported.

## Data Availability

All the necessary data have been presented in the manuscript and tables.
